# The complete chloroplast genome and phylogenetic analysis of *Artocarpus champeden*

**DOI:** 10.1080/23802359.2021.1987165

**Published:** 2021-10-07

**Authors:** Ying-Feng Niu, Jin Liu

**Affiliations:** Yunnan Institute of Tropical Crops, Xishuangbanna, China

**Keywords:** Chloroplast genome, phylogenetic analysis, *Artocarpus champeden*

## Abstract

*Artocarpus champeden* Spreng. is a popular fruit tree, grown in tropical and subtropical regions. Besides food, *A. champeden* is also a medicinal plant with various medicinal properties. In this study, *A. champeden* chloroplast genome was sequenced, assembled, and annotated due to its rich information on species evolution and inter-species genetic relationships. The quadripartite structure of *A. champeden* complete chloroplast genome is 158,568 bp in length and comprises a large single-copy region (LSC) of 88,076 bp, a small single-copy region (SSC) of 19,028 bp, and two inverted repeat regions (IRa and IRb) of 25,732 bp. A total of 131 genes were annotated, including 85 protein-coding genes, 37 tRNA genes, eight rRNA genes, and one pseudogene. Phylogenetic analysis revealed a close relationship between *A. champeden* and *A. heterophyllus*. In addition, the study provides abundant genomic information for future phylogenetic studies of *A. champeden* and the Moraceae family.

*Artocarpus champeden* (Lour.) Spreng. (*A. integer* (Thunb.) Merr. 1917) is a popular fruit tree belonging to the family Moraceae (Widyawaruyanti et al. 2007). It is traditionally grown in tropical and subtropical regions, particularly in Southeast Asian countries, including Thailand, Peninsular Malaysia, Myanmar, Vietnam, and Indonesia (Syah et al. 2006; Lim et al. 2011). Both the flesh and seeds of *A. champeden* fruits are edible. The seeds are a good source of dietary fiber and resistant starch (Zabidi and Aziz 2009). Moreover, the wood of *A. champeden* is used commercially as timber (Achmad et al. 1996).

*Artocarpus champeden* is also a medicinal plant with various medicinal properties, including antimalarial, anticancer, and cell adhesion activities (Lopes et al. 2018). The seeds contain lectins such as IgA1-reactive and D-galactose-binding lectin. These lectins are used in biomedical research to detect tumors and identify glycoproteins (Lim et al. 1997). The wood pulp of *A. champeden* has essential bioactive compounds, including ascorbic acid and total phenolics (Lopes et al. 2018). Several isoprenyl flavonoids with cytotoxicity (Hakim et al. 1999) and antimalarial activity (Boonlaksiri et al. 2000) have been isolated from *A. champeden*.

While the economic potential of *A. champeden* is due to its food and medicinal value, very little research on its genome exists. Chloroplast genomes are rich in genetic information and are fundamental in evolutionary studies. This is the first study to sequence, assemble, and annotate the chloroplast genome of *A. champeden*.

*Artocarpus champeden* was collected from the Xishuangbanna Tropical Flowers and Plants Garden (100.785968 E, 22.012288 N), and the genomic DNA was extracted using the DNeasy Plant Mini Kit (Qiagen). The specimen and DNA were deposited in the herbarium and cryogenic sample library of the Yunnan Institute of Tropical Crops (http://www.yitc.com.cn, Dr. Jin Liu, liujin06@126.com) with voucher numbers YITC-2020-FZ-M-028 and D2020-FZ-M-028, respectively. Paired-end (PE) sequencing was conducted using the Illumina HiSeq 2500 platform (Illumina, San Diego, CA, USA) with 350 bp library insert sizes. The clean reads were assembled with SPAdes-3.5.0 (http://soap.genomics.org.cn/soapdenovo.html) based on sequence overlap and paired-end relationships. Kmer 79 and Kmer 97 values were used, and Sanger sequencing verified the four boundaries of the IR region. Annotation was performed by CpGAVAS2 (Shi et al. 2019) and ORF Finder (https://www.ncbi.nlm.nih.gov/orffinder/). The chloroplast genome data of *A. champeden* was submitted to GenBank (http://www.ncbi.nlm.nih.gov/) under the accession number MT900597.

The quadripartite structure of *A. champeden* whole chloroplast genome is 158,568 bp in length, and comprises 49,698 A bases (31.34%), 51,343 T bases (32.38%), 28,466 G bases (17.95%), and 29,061 C bases (18.33%). Moreover, it contains a large single-copy region (LSC) of 88,076 bp, a small single-copy region (SSC) of 19,028 bp, and two inverted repeat regions (IRa and IRb) of 25,732 bp. The total GC content of the whole chloroplast genome of *A. champeden* is 36.28%, with corresponding values of the LSC, SSC, and IR regions of 33.96%, 29.47%, and 42.76%, respectively. In total, 131 genes, including 85 protein-coding genes, 37 tRNA genes, 8 rRNA genes and 1 pseudogene, were annotated.

Chloroplast genome sequences of 22 species (Figure 1) of Moraceae family were downloaded from GenBank and used to study the phylogeny of *A. champeden*, Multiple sequence alignment was performed using MAFFT (Katoh and Standley 2013), The jModelTest 2.1.7 (David 2008) software was employed to analyze nucleotide substitutions model under the Akaike Information Criterion (AIC), the GTR + G + I model was selected for nucleotide and the maximum-likelihood analysis was conducted with RAxML8.2.4 (Stamatakis 2014). *Eriobotrya malipoensis,* which belongs to the Rosaceae family, was the out-group, and the node support was estimated from the results of 1000 bootstrap replicates. The phylogenetic analysis revealed a close relationship between *A. champeden* and *A. heterophyllus* (Figure 1). This study provides abundant genomic information for future phylogenetic studies of the Moraceae family.

**Figure 1.**
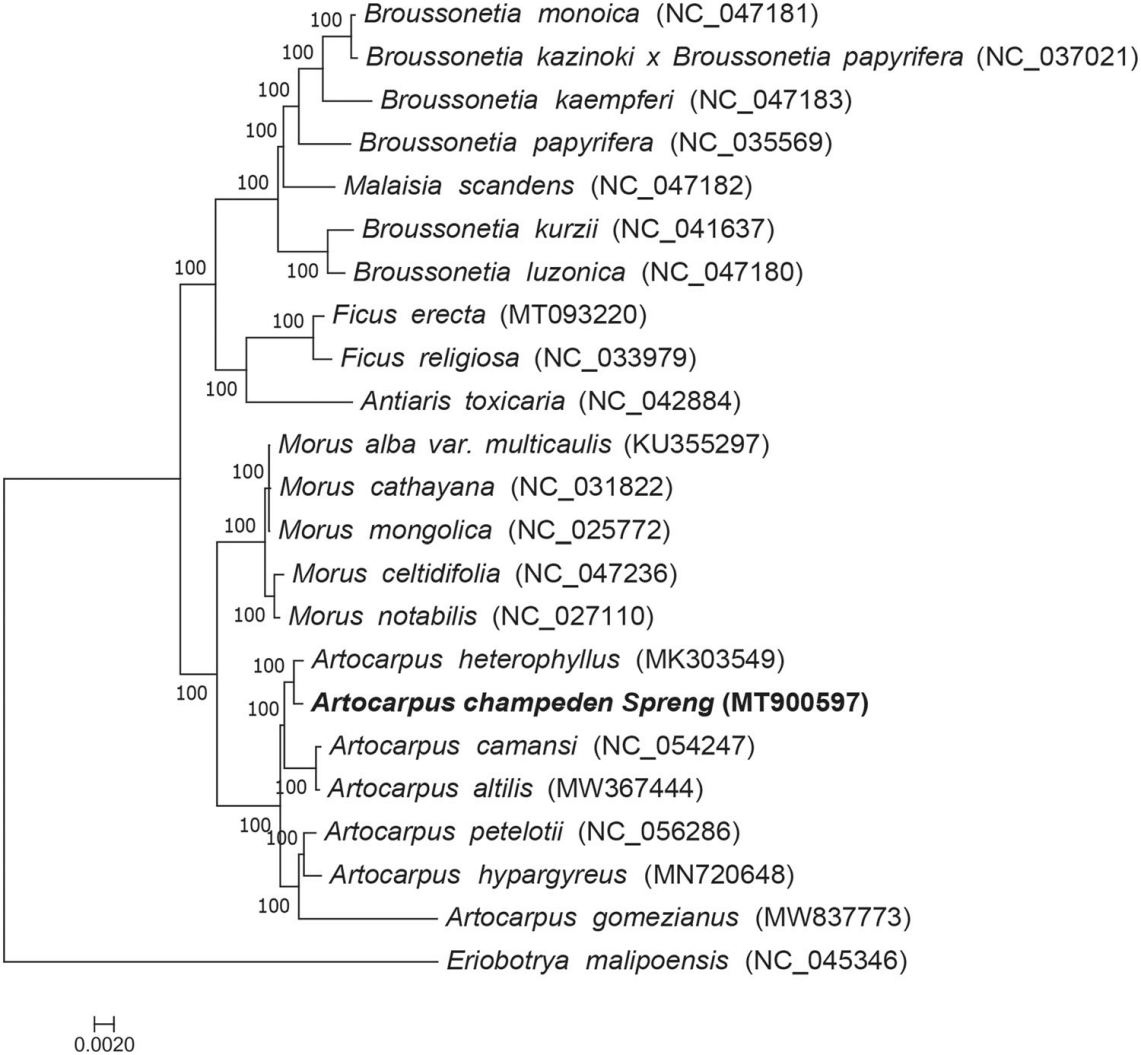
Maximum-likelihood tree based on the complete chloroplast genome sequences of *A. champeden* and 21 other species of the Moraceae family. *Eriobotrya malipoensis,* which belongs to the Rosaceae family is the out-group.

## Data Availability

The genome sequence data that support the findings of this study are openly available in GenBank of NCBI at (https://www.ncbi.nlm.nih.gov/) under the accession no. MT900597. The associated BioProject, SRA, and Bio-Sample numbers are PRJNA703082, SRR13781753, and SAMN18011290 respectively.
